# Prevalence of Depression and Anxiety among Patients with Multiple Sclerosis Attending the MS Clinic at Sheikh Khalifa Medical City, UAE: Cross-Sectional Study

**DOI:** 10.1155/2015/487159

**Published:** 2015-07-09

**Authors:** Taoufik Alsaadi, Khadija El Hammasi, Tarek M. Shahrour, Mustafa Shakra, Lamya Turkawi, Abdulla Mudhafar, Lina Diab, Mufeed Raoof

**Affiliations:** ^1^Department of Neurology, Sheikh Khalifa Medical City (SKMC), Abu Dhabi, UAE; ^2^Department of Psychiatry, Sheikh Khalifa Medical City (SKMC), Abu Dhabi, UAE

## Abstract

Depression and anxiety are reported to be prevalent in patients with MS, with prevalence rates ranging from 20% to 50%. Unfortunately, the rates, patterns, and risk factors are not well studied in our Middle East region, and, to our knowledge, not at all in UAE. Using standardized screening tools, we observed that 17% and 20% of 80 patients seen in MS clinic had scores consistent with major depression and anxiety disorders, respectively, at a rate that was not statistically different when compared to age and sex matched controls. None of the studied variables including duration of the disease, EDSS scores, age, gender, and the level of education had any significant correlation with the rates of both disorders. Almost two-thirds of the patients with scores consistent with major depression and anxiety were not on antidepressant and antianxiety medications.

## 1. Introduction

MS is a chronic immune-mediated disease of the central nervous system that typically begins in early adulthood and often leads to long-term disability [1]. The lifetime risk of major depression in people with MS has been estimated to be as high as 50% compared to 10 to 15% in the general population [2, 3]. Furthermore, depression has a negative impact on patients with MS (PWMS) on several domains. It is well established that depression is one of the main determinants of quality of life (QOL) in patients with MS [4] and by itself, and, especially, when combined with anxiety, it increases the risk of suicidality in these patients [5, 6]. Finally, depression often impairs relationships and reduces adherence to disease modifying treatments [7, 8].

The findings from all these studies underscore the importance of recognizing mood disorders in MS and call for the need to effectively screen and treat these disorders. However, despite its relative high frequency, great impact on the QOL, and overall care for PWMS, depression and anxiety remain undertreated and unrecognized in a significant percentage of these patients [9]. This finding comes as a result of the increasing demands in any busy neurology practice to see more and spend less time with patients. It is, therefore, very likely in that setting to miss the symptoms of depression and anxiety.

Several instruments have been used to screen for depression and anxiety in PWMS; some of them are time consuming in a busy clinic setting. The Patient Health Questionnaire nine-item (PHQ-9) depression scale, which is used in this study, is a brief, self-administered tool for the screening and diagnosis of depression. It is based on the nine DSM-IV criteria of depression and it is quite sensitive to change in depression scores over time and can be used to monitor response to therapy in this population [10]. The PHQ-9 depression scale has been validated in the general population, indicating a prevalence rate of 9.2% for a current depressive disorder [11, 12]. As a matter of fact, a recent study has compared this tool to other widely used tools in MS research and concluded that PHQ-9 by providing a tentative cut-off score is quite sensitive and specific for identifying patients with major depressive disorder [13]. Clearly, the use of this and any other screening instruments for psychiatric research in MS should be followed by structured psychiatric interviews to confirm the diagnoses. Similarly, and to screen for anxiety disorders, the Generalized Anxiety Disorder 7 (GAD-7) scale was adopted. It consists of a brief, 7-item questionnaire that takes less than 3 min to complete, unlike the Hospital Anxiety and Depression Scale (HADS), which is a 14-item self-report screening scale that takes longer time to complete. A score of >9 in GAD-7 is indicative of a Generalized Anxiety Disorder (GAD). This valuable instrument is used to screen for anxiety in primary care patients [14]. Furthermore, a recent study supports the reliability and internal validity of the GAD-7 for use in MS [15].

In this study, we sought to determine the prevalence rates of both depression and anxiety in our MS population attending the MS clinic at our tertiary referral center and to compare these rates to healthy age and sex matched controls. We have also examined whether there was a difference in depression and anxiety rates as a function of patients' socioeducational related variables, MS duration, the type of immunomodulators, and the scores on EDSS.

## 2. Materials and Methods

All consecutive patients attending the MS clinic over a 4-month period, from October to February 2015, at Sheikh Khalifa Medical City (SKMC), were asked to enroll in this study. MS clinic in SKMC is one of the two major tertiary referral clinics in the 1.5 million population of the Emirates of Abu Dhabi serving close to 400 patients with MS. Only three patients have declined to participate. Those who agreed to participate were asked to complete the PHQ-9 and GAD-7 questionnaires. In this study, we used the self-administered English versions of PHQ-9 and GAD-7, and, for our few Arabic speaking patients only attending our clinic, we used the Arabic validated versions of both tools [16]. The patients completed the questionnaires before their assessment in the consultation room. We included patients with a confirmed diagnosis of relapsing remitting MS, as per the revised McDonald 2010 criteria, older than 18 years of age that either were seen for the first time in this clinic or were seen for a follow-up visit [17]. We excluded patients with progressive cognitive deficit that may render patients incapable of signing the research consent form. The control group included a comparable number of healthy people matched with our patients group for age and sex, Table 1. These controls were randomly selected from families of patients visiting the hospital clinics, college and university students, employees of nonhealth related companies, and random people visiting the malls. Only two of our approached controls have declined to participate in the study. This study was approved by the local institutional review board.

Age, gender, number of relapses in the 6 months prior to the clinic visit, current immunomodulators, EDSS scores, MS duration, and PHQ-9 and GAD-7 scores were all recorded. Marital status and education level were recorded by completing a validated Brief-WHO QOL questionnaire by all patients.

In this study, we classified patients with PHQ-9 and GAD-7 scores ≥ 10 as having major depression and anxiety disorder, respectively. Data analysis was performed using the Statistical Package for Social Sciences (SPSS, version 21). Descriptive statistics was used for demographic data to show the respondents' characteristics. Chi-square (*χ* ^2^) test was used to assess the relationship between both depression in PHQ-9 (cut-off point 10) and the categorical data and anxiety in GAD-7 (cut-off point 10) and the categorical data. Bivariate analysis was used to examine correlations between different variables and both depression in PHQ-9 (cut-off point 10) and anxiety in GAD-7 (cut-off point 10). As both depression and anxiety were on a categorical scale, we used Spearman's Rho correlation coefficient (*r*) to determine the correlation of both depression and anxiety as dependent variables and various independent demographic and clinical characters.

## 3. Results

A total of 80 patients were seen over the 4-month period. The mean age was 35 years (SD 10.60). There were 52 female (65%) and 28 (35) male patients, Table 2. There was no gender difference in PHQ-9 scores (*P* = 0.459) and in GAD-7 scores (*P* = 0.131). Similarly, age, level of education (high school versus college), and marital status did not reach statistical difference on both scores, Tables 3(a) and 3(b).

A total of 14 out of the 80 patients (17.6%) reported PHQ-9 scores ≥ 10 consistent with major depression. As for anxiety scores, 16 patients (20%) had scores ≥ 10 consistent with anxiety disorder. On the other hand, 16% and 15% of age and sex matched controls had scores consistent with depression and anxiety, respectively. When compared to our MS population, there were no statistical differences between the 2 groups with *P* values 0.83 and 0.408, respectively, and OR (1.09: 0.48–2.50) and 1.41 (0.62–3.22), respectively, Table 4.

Nine of our fourteen patients who were classified as having major depression were not on antidepressant medications at the time they completed the PHQ-9. However, mean depression scores were not significantly higher (*P* = 0.070) for patients who were on antidepressant medication at the time of the clinic visit (7.77), as compared with patients not taking antidepressant medication (4.60). The main effect for MS duration and the number of MS attacks in the previous 6 months did not reach any statistical significance on either depression or anxiety rates (*P* = 0.4, 0.49/*P* = 0.17, 0.51). Similarly, we examined whether depression and anxiety scores varied as a function of being on immunomodulators versus not being on immunomodulators. This variable also did not correlate with either (*P* = 0.499, 0.638, resp.), Tables 3(a) and 3(b). Furthermore, the median EDSS score of our cohorts is 0.5, whereas the mean score is 1.74 (SD 2.21). We examined whether depression and anxiety scores varied as a function of EDSS scores. This variable also did not correlate with either (*P* = 0.510, 0.765, resp.), Tables 3(a) and 3(b).

## 4. Predictors of Depression and Anxiety among Patients with Multiple Sclerosis

Using the multiple logistic regression analysis with depression at cut-off point 10 or more with PHQ-9 as the dependent variable and the significant factors leading to depression as independent variables (prescribed antidepressant, PHQ-9 scores, GAD-7 score, and being anxious, Table 3(a)), the results show that only the score of PHQ-9 was statistically significant (Wald = 10.418, *P* = 0.001, 95% CI 1.361–3.527) as a predictor of depression in PWMS. Further logistic regression analysis for anxiety symptoms at cut-off point 10 or more, with GAD-7 is the dependent variable, and the significant factors leading to anxiety are independent variables, show that no factors are significantly predicting anxiety in that model.

## 5. Discussion

To our knowledge, our pilot study was the first to report the rates of mood disorders among PWMS in our region. The rates of depression and anxiety in our cohorts are similar to some of the previously reported figures in other studies [16, 17]. Other studies, on the other hand, have reported higher figures. The variability of rates among the above studies can be partly due to differences in study design and settings, as studies from MS clinics, community samples, or administrative health database are expected to have different rates [18]. Furthermore, the differences in the utilized tools to screen for both disorders among all the studies and the fluctuating course of MS somatic symptoms may be other contributing factors. It is worth pointing out, however, that rates of depression and anxiety vary among studies reporting point prevalence versus studies reporting lifetime prevalence [19]. In a study reporting point prevalence of depression in a large community sample utilizing PHQ-9 tool, similar to ours, a higher rate of depression of 26% was reported [20]. On the other hand, no studies have reported point prevalence of anxiety rates using GAD-7 tool, which has been used in the current study.

Contrary to other studies, PWMS in our populations were at a similar risk of having depression and anxiety rates as compared to the general population. Previous reports utilizing larger sample studies and utilizing other screening tools or following structured psychiatric interviews have indicated several fold increase in the risk of depression and anxiety when compared to the general population [19, 21, 22]. This negative and unexpected association, unlike previous studies, may indeed reflect a true lack of association in our population. On the other hand, this negative finding can be explained by several factors. Our small sample size, as compared to the larger reported samples in other studies, may in part explain this dissociation. For example, a large UK study has utilized a web based data that enrolled more than 4000 patients and concluded that the risk of depression was close to 50% [23]. In addition, most of our enrolled patients did not have a relapse at the time of screening. A previous study has shown almost twice the risk of depression rates at the time of relapse when compared to screening 2 months following a relapse [24]. Furthermore, it is well known that differences in the rates of mood disorders are quite common in the MS patients given the heterogeneity of the population and the diversity of symptoms which could also account for our negative results. Finally, genetic, environmental, and vitamin D status are other factors that we did not control for in our study, which could have an impact on the rates of both disorders [21, 25].

In the current study, and on a separate note, the lack of correlation between EDSS scores, duration of MS, and all the other study variables and the rates of both depression and anxiety can be well understood given the heterogeneity of the populations and the variability of symptomatology, the rates, and degree of disability among this population. Notably, the median EDSS score in our patients was 0.5, reflecting a mildly disabled population, which could explain the relatively lower rates of depression and anxiety in our cohorts as compared to the reported higher rates in other studies. However, previous studies have shown a strong correlation between EDSS scores and the rates of mood disorders. Indeed, a recent study has shown that patients with EDSS scores less than 3, similar to our cohorts, were at least twice less likely to suffer from depression and/or anxiety [26]. On the other hand, other studies concluded the opposite [18].

## 6. Conclusions

We realize the limitations of our study being a single center and probably a small and underpowered size study. However, we believe that a larger and powered study from our region is needed to confirm our finding. Still, and as previously demonstrated in several studies, the robust negative impact of both depression and anxiety on PWMS overall QOL should call for careful screening for these disorders in this population. Indeed, less than one-third of our patients were on antidepressants and antianxiety medications at the time of the study. These figures reflect the increased pressure on neurologists to see more but spend less time with patients at clinics. Therefore, it is expected that the development of time efficient screening tools would allow busy centers to adopt the practice of screening for mood disorders at the waiting area prior to patient's evaluation by the neurologist.

## Figures and Tables

**Table 1:** Characteristics of controls and MS group.

	MS patients (*n* = 80)	Control (*n* = 80)
Age (mean)	35	34.1
Female	52 (65%)	51 (63.8%)
Male	28 (35%)	29 (36.2%)
Prevalence of depression	17.5%	16.2%
Prevalence of anxiety	20%	15%

**Table 2:** Sociodemographic information and disease related feature.

	Total (*n* = 80) %	Range	Mean (SD)
Age (years)	—	18–65	35.1 (10.5)
Sex			
Females	52 (65%)	—	—
Males	28 (35%)		
Marital status			
Married	50 (62.5%)	—	—
Not married	30 (37.5%)		
Education			
Lower education	33 (41.3%)	—	—
Higher education	47 (58.7%)		
Multiple sclerosis duration since diagnosis (years)	—	0.1–23	7.8 (4.6)
EDSS score		0–6.5	1.7 (2.2)
			Median (0.5)
Patients relapsed within 6 months			
No relapse	74 (92.5%)	—	—
Relapse	6 (7.5%)		
Treatment status			
On treatment	54 (67.5%)	—	—
Not on treatment	26 (32.5%)		

**(a):** 

Bivariate analyses		
Variable	*r*	*P* value
Gender	.084	0.459
Age (mean, SD)	−.067	0.557
Duration of MS	−.125	0.268
Marital status	.174	0.123
Education	.142	0.208
EDSS score in the past 6 months	−.075	0.510
MS treatment status	−.077	0.499
Prescribed antidepressants	.222	0.047
PHQ-9 scores	.721	0.0001
GAD-7	.587	0.0001
Anxiety (score 10 or more in GAD-7)	.560	0.0001

**(b):** 

Bivariate analyses		
Variable	*r*	*P* value
Gender	.170	0.131
Age (mean, SD)	.084	0.461
Duration of MS	.048	0.672
Marital status	.129	0.254
Education	.025	0.823
EDSS Score in the past 6 months	.034	0.765
MS treatment status	.053	0.638
Prescribed antianxiety medications	.203	0.070
PHQ-9 scores	.678	0.0001
GAD-7	.855	0.0001
Depression (score 10 or more in PHQ-9)	.560	0.0001

**Table 4:** MS and control group.

Disorder	MS group *N* (%)	Control *N* (%)	OR (95% CI)	*P* value
Depression				
Present	14 (17.5%)	13 (16.2%)	1.09 (0.48–2.50)	0.83
Absent	66 (82.5%)	67 (83.8%)		
Anxiety				
Present	16 (20%)	12 (15%)	1.41 (0.62–3.22)	0.408
Absent	64 (80%)	68 (85%)		
